# Lymphoma Presenting as a Soft Tissue Mass

**DOI:** 10.5334/jbsr.2893

**Published:** 2022-10-11

**Authors:** Joana Granadas, Marta Baptista, Sérgio Ferreira

**Affiliations:** 1 Hospital Prof. Doutor Fernando Fonseca, PT

**Keywords:** Lymphoma, Musculoskeletal system, MRI

## Abstract

**Teaching Point:** Lymphomatous involvement of the skeletal muscle is rare; however, because lymphoma can be treated with chemotherapy, it is crucial to consider it in the differential diagnosis of a soft tissue mass in the appropriate clinical and imaging setting.

## Case History

A 37-year-old man presented to the emergency department with a one-month history of non-traumatic left knee pain and swelling. His previous medical history was irrelevant. Physical examination revealed a tumefaction on the left medial thigh. Laboratory tests were unremarkable.

**Figure 1:**
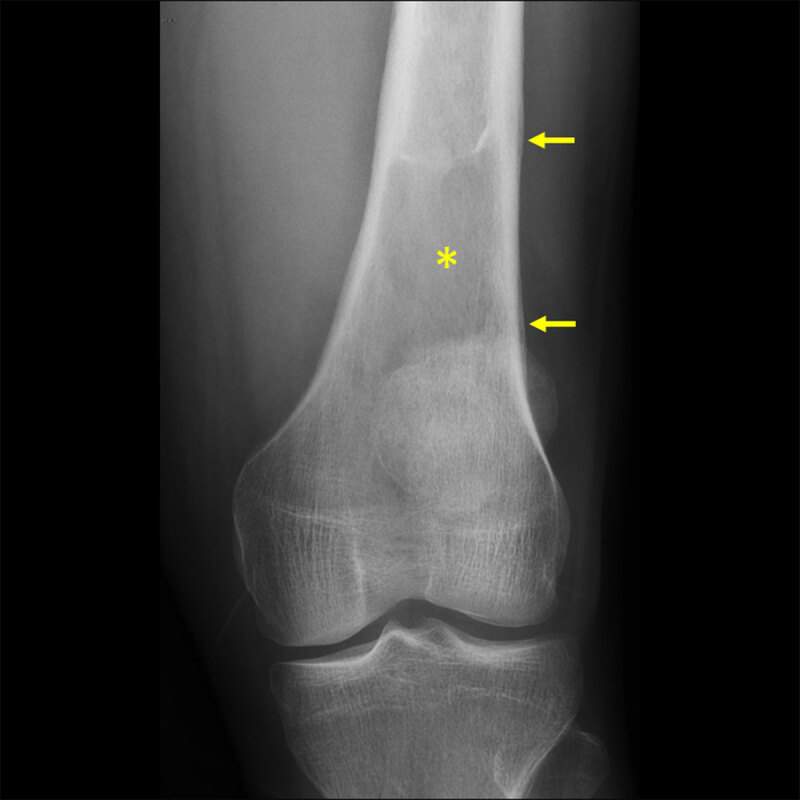
Knee radiograph showing a lytic lesion in the femur with lamellated periosteal reaction.

**Figure 2:**
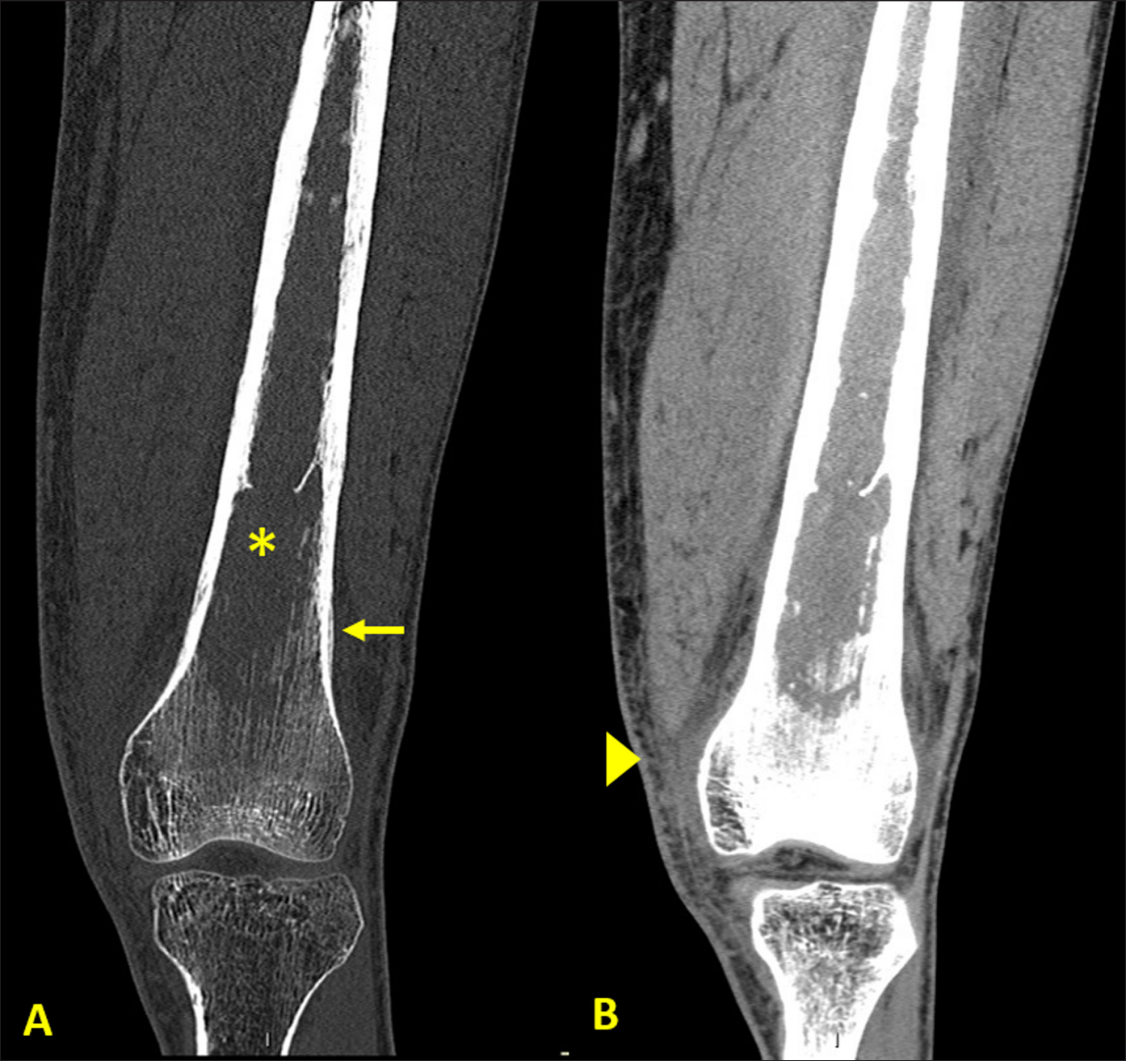
CT of the knee showing preserved integrity of the femur’s cortex.

The patient underwent a knee radiograph that showed a lytic bone lesion in the femur (asterisk in Figure 1) with lamellated periosteal reaction (arrows in Figure 1). Computed tomography (CT) of the knee showed loss of the normal trabeculation of the femur’s medulla (asterisk in Figure 2A), with preserved integrity of the cortex. Periosteal reaction (arrow in Figure 2A) and densification of the adjacent soft tissues (arrowhead in Figure 2B) were noticed.

**Figure 3:**
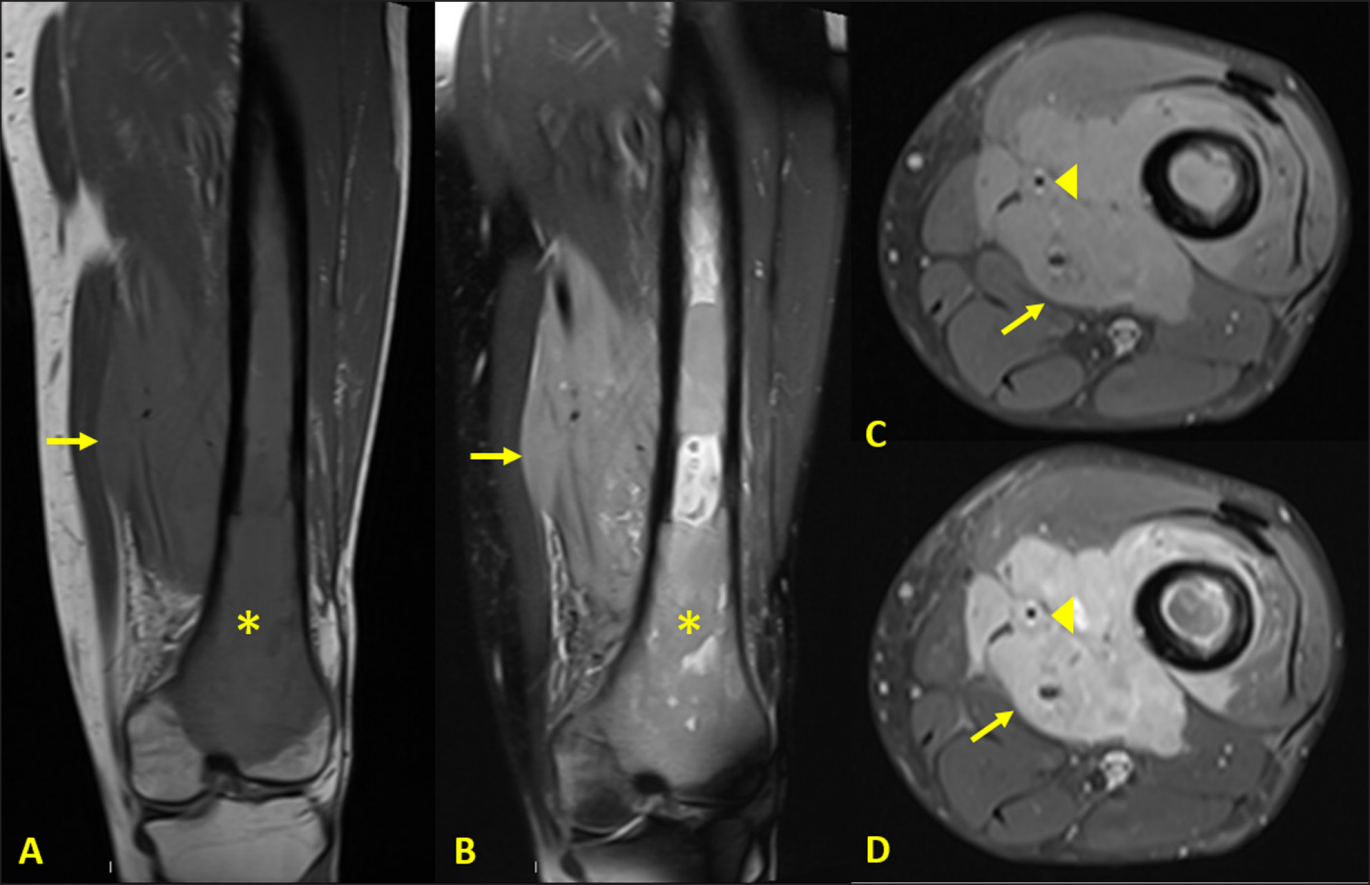
MRI of the knee showing a large soft tissue mass infiltrating the thigh’s muscles.

Magnetic resonance imaging (MRI) revealed a large soft tissue mass infiltrating the thigh’s muscles (arrows in Figure 3) and the neuro-vascular bundle (arrowheads in Figure 3C and 3D). It had an intermediate signal on T1-weighted images (T1WI) (Figure 3A and 3C), high signal on DP-weighted images with fat saturation (FS DPWI) (Figure 3B) and enhanced after contrast injection (Figure 3D). The medulla of the femur was hypointense on T1WI (asterisk in Figure 3A) and hyperintense on FS DPWI (asterisk in Figure 3B). There was no cortical disruption.

The biopsy revealed a B-cell non-Hodgkin’s lymphoma.

Chest-abdomen-pelvis CT showed adenopathies on the left external iliac and inguinal regions. There were no other affected organs or lymph node chains. He was treated with chemotherapy and the disease regressed completely.

## Comment

Lymphoma can affect the musculoskeletal system either as a disseminated process or as an isolated disease. Lymphomas of the skeletal muscles are mostly non-Hodgkin’s lymphomas and occur predominately in the thighs, chest, and arms. Patients may present with B symptoms and high LDH serum levels [1].

A wide variety of imaging modalities may be used, and each offers different details about the disease. However, MRI is better at defining anatomical boundaries and the internal architecture of soft tissue. It shows an intermediate signal on T1WI, and high signal on T2WI (relative to muscle). Contrast enhancement can be either homogeneous or heterogeneous [1].

Imaging findings can be variable. Multiple muscles may be involved, and it can manifest as a diffuse or focal muscular enlargement, an infiltrative lesion, or a focal mass. In cases of tumor multifocality, involvement may be continuous or discontinuous. It may not be circumscribed by the muscle compartment and fascial planes [1].

Distinguishing lymphoma from other neoplastic and non-neoplastic disorders can be challenging. Although relative cortical preservation in the context of a sizable extraosseous tumor is not specific, it is a useful finding for considering lymphoma in the differential diagnosis [1].
